# Novel use of the Nuwellis® dual extended length catheter (dELC) as umbilical venous access for neonatal kidney replacement therapy: case series

**DOI:** 10.1007/s00467-026-07363-x

**Published:** 2026-05-18

**Authors:** Kara Short, Alyssa Umberger, Meggie Mathis, Mallory Walker, Jacki Harmon, Lyndsay Harshman, Brynna Van Wyk, Kyle Merrill

**Affiliations:** 1 Pediatric and Infant Center for Acute Nephrology, Children’s of Alabama, Birmingham, AL USA; 2 Neonatal Intensive Care Unit, Children’s of Alabama, Birmingham, AL USA; 3 University of Iowa Healthcare Stead Family Children’s Hospital, Iowa City, IA USA; 4 The Division of Pediatric Nephrology at University of Alabama at Birmingham, Park Place 202, 1600 5th Avenue South, Birmingham, AL 35233 USA

**Keywords:** CKRT, Continuous kidney replacement therapy, Neonatal, Critical care, Dialysis, Vascular access

## Abstract

**Background:**

Neonates with congenital kidney failure or acute kidney injury often require continuous kidney replacement therapy (CKRT). Historically, venous access for CKRT in this population has relied on non-approved adult catheters, commonly via the internal jugular (IJ) vein. Limited international reports describe using the umbilical vein for CKRT, but this practice is not widespread in the USA. The Nuwellis® dual lumen extended length catheter (dELC), originally created for adult ultrafiltration, may support alternative access for neonatal CKRT.

**Methods:**

This case series describes the clinical course of four neonates requiring CKRT using a 6 French Nuwellis® dELC placed in the umbilical vein at University of Iowa Stead Family Children’s Hospital and Children’s of Alabama. Patient demographics, CKRT prescriptions, access characteristics, and complications were reviewed.

**Results:**

All four neonates underwent successful umbilical vein placement of the 6 Fr dELC. Weight at initiation ranged from 2.23 to 4.1 kg, with blood flow rates 20–40 mL/min. Epoprostenol anticoagulation was used in three patients. Half of the cohort later transitioned to a tunneled catheter, and one transitioned to an IJ catheter following fluid removal. One catheter developed hub cracks likely related to lumen caps, and one patient experienced flow limitations attributed to positioning rather than catheter function. Two patients had no catheter-related complications.

**Conclusions:**

Umbilical venous access using the 6Fr dELC may represent a viable alternative for neonatal CKRT. Coil reinforcement within the catheter may support patency and better functionality. Additional studies are needed to assess safety and broader applicability.

**Graphical abstract:**

A higher resolution version of the Graphical abstract is available as Supplementary information

**Figure:**
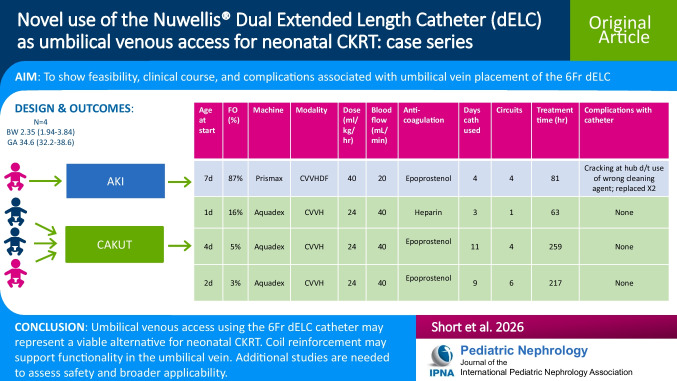

**Supplementary Information:**

The online version contains supplementary material available at 10.1007/s00467-026-07363-x.

## Introduction

Vascular access remains a critical and challenging aspect of delivering continuous kidney replacement therapy (CKRT) in neonates with kidney failure [1]. The technical demands are heightened by the small vessel size, limited blood volume, and the need for reliable, high-flow access to support extracorporeal therapies [2]. According to the National Kidney Foundation’s Kidney Disease Outcomes Quality Initiative (KDOQI) guidelines, pediatric patients—particularly neonates requiring unplanned dialysis—often need central venous catheters (CVCs) as a temporary bridge to permanent access or until a peritoneal dialysis (PD) catheter can be placed [3]. However, CVCs are associated with increased risks of bloodstream infection, central venous stenosis, and long-term morbidity, underscoring the importance of minimizing their use and duration whenever feasible [3, 4].

A major limitation in the USA is the lack of commercially available small gauge dual lumen dialysis catheters, necessitating the adaptation of adult or pediatric devices for neonatal use [4–6]. This often requires technical modifications, such as shortening catheter length or using smaller French sizes, which may compromise flow rates and increase the risk of complication [4]. Recent studies have explored alternatives, such as the use of small tunneled CVCs (e.g., 6 Fr), which have demonstrated longer functional duration and fewer revisions compared to standard hemodialysis catheters, albeit with a higher infection rate [7]. This, coupled with the development of low-volume circuits and infant-specific platforms, is beginning to address these barriers [5, 6, 8].

Case studies and small series have described the use of a hemodialysis catheter placed in the umbilical vein for neonates receiving CKRT, particularly in neonates or infants less than 5 kg or when alternative access is not feasible [9]. The umbilical vein provides a readily accessible route in the immediate neonatal period, allowing for adequate blood flow rates required for CKRT initiation. For example, Kaneko et al. reported successful CKRT with rapid ammonia clearance in an extremely low birth weight (ELBW) infant with hyperammonemia using the umbilical vein for vascular access, demonstrating rapid ammonia clearance and technical feasibility, though noting the challenges related to the immaturity of the patient and the risks inherent to this approach [10].

Overall, this approach has been reported as a feasible but high-risk option, reserved for situations without alternative options due to significant infectious and vascular risks [10, 11]. The risks associated with CVC placement in the umbilical vein include increased risk of infection due to the high colonization of the umbilical stump, malposition of the catheter, and increased risk for thrombosis in this vessel specifically [11, 12].

This paper evaluates the six French (Fr) 11-cm Nuwellis dual lumen extended length catheter (dELC) used in the umbilical vein as a temporary option for neonatal CKRT, particularly in cases where traditional access is not feasible. We highlight technical considerations, blood flow capability, and early clinical performance. Given the ongoing challenge of securing reliable access in this population, the dELC offers a promising alternative, though its risks and limitations must be carefully considered in critically ill neonates.

## Methods

This is a retrospective case series describing the clinical course of four neonates at two tertiary pediatric centers: the University of Iowa Health Care Stead Family Children’s Hospital (SFCH) and Children’s of Alabama (COA), who required CKRT using the Nuwellis® 6 French 11-cm dELC placed via the umbilical vein during the neonatal period.

The dELC is a venous access device created for use with the Aquadex machine. It has features similar to traditional hemodialysis catheters, with separated infusion and withdrawal openings. Its stainless-steel coil reinforcement offers increased stability of the small 16-gauge lumens. The catheter is intended for peripheral insertion into the antecubital fossa in adult patients. It is made to tolerate blood flows of 40 mL/min, which is the max blood flow for the Aquadex device.

Placement of the dELC in the umbilical vein requires experience and knowledge of the standard approach to placing a CVC in the same vessel. All patients had CVCs prior to arriving at their respective centers. In a sterile technique, the umbilical CVC sutures were cut, the umbilical vein was accessed using forceps or dilator, and the dELC was advanced to the estimated length in cm, calculated by birth weight × 3 + 9. Placement is confirmed by chest X-ray [13].

Demographic variables including sex, gestational age, and birth weight were collected. Clinical characteristics evaluated included etiology of kidney dysfunction, fluid overload at CKRT initiation, and hemodynamic status. Technical aspects of CKRT were reviewed, including device platform (Aquadex (Nuwellis Inc, Eden Prairie, MN, USA) or PrisMax (Vantive, Deerfield, IL, USA)), modality (CVVH or CVVHDF), blood flow rate, anticoagulation strategy, effluent dosing, and vascular access details. Outcomes of interest included catheter-related complications, circuit survival hours, number of circuits used, duration of therapy, and patient disposition. Data were abstracted from electronic medical records and summarized descriptively.

## Case series

### Patient A

Patient A is a female infant thirty-two weeks four days gestation at 1.935 kg with APGARs of 1, 6, and 8 at an outside facility. She was intubated after developing respiratory distress and severe metabolic acidosis on day of life (DOL) 1. She was transferred to the pediatric cardiac intensive care unit (PCICU) at SFCH due to concerns for left ventricular heart failure as well as acute liver failure with coagulopathy. She developed stage 2 AKI on DOL 5 and oliguria, which was treated with medical management, developing worsening fluid overload to 50%. We attempted initial treatment with Aquadex for slow continuous ultrafiltration (SCUF) via peripheral access with power injectable IVs; however, adequate flows could not be obtained. We were unable to obtain traditional CKRT access in the jugular or femoral veins due to edema. Ultimately, the decision was made to pursue the use of the dELC via the umbilical vein for venous access.

She underwent catheter placement by interventional radiology on DOL 6. Due to the need for ultrafiltration and clearance, she was initiated on CKRT via the PrisMax device, as we do not perform continuous veno-venous hemofiltration (CVVH) using the Aquadex device at our institution, with a blood prime and the HF20 circuit. On the day of CKRT initiation, she was 3.62 kg and 87% fluid overloaded by weight. She was placed on continuous veno-venous hemodiafiltration (CVVHDF) at a blood flow rate of 20 mL/min (10 mL/kg/min by dry weight) per treatment team. CKRT was anticoagulated with epoprostenol anticoagulation starting at a rate of 2 ng/kg/min and titrated up by 2 ng/kg/min as needed for circuit life to a maximum dose of 8 ng/kg/min. CKRT dosing was 40 mL/kg/h (700 mL/h/1.73 m^2^). She was treated with 4 circuits over 4 days for a total of 81 h of therapy. Circuit life for each circuit was 5, 27, 39, and 10 h respectively. Overall, the circuit life was mostly affected by issues with cracking of the dialysis catheter hub, thought to be due to the use of alcohol cleansing and capping of the catheter while the catheter was wet, but it is not clear as all care was per manufacturer guidelines and similar to other institutions. After this time, the patient’s weight decreased to 3.38 kg and the ICU team was able to successfully place a 7 Fr non-tunneled right IJ line to continue CKRT due to improvement in edema allowing placement.

She continued CKRT for 17 days. Ultimately, she was unable to tolerate dialysis due to continued hypotension refractory to medical management, and the family decided to redirect to comfort care.

### Patient B

Patient B was a di-chorionic/di-amniotic twin male born at thirty-two weeks two days gestation via cesarean section for non-reassuring fetal heart tones (NRFHT), with a birth weight of 2000 g, APGARs 1, 2, 5, and 7, and suspected lower urinary tract obstruction (LUTO). He required resuscitation and intubation for pulmonary hypoplasia. He was transferred to COA’s neonatal intensive care unit (NICU) for anuria on DOL 0. He was deemed too unstable to go to the operating room to place a tunneled dialysis catheter for CKRT, as is our institution’s practice for all neonates needing CKRT, so the neonatal nurse practitioner (NNP) replaced his central line in the umbilical vein with the dELC. CVVH via Aquadex was initiated at a weight of 2.23 kg and 11.5% fluid overload on DOL 1.

Blood flow was maintained at 40 mL/min with systemic heparin anticoagulation, starting at 20 u/kg/h, titrating to aPTT goal of 50–70 s. CVVH dosing was prescribed 24 mL/kg/h (172 mL/h/1.73 m^2^). Circuit survival was 63.2 h with a single circuit utilized. CKRT treatment was uneventful, with no reported complications during the 3-day course of therapy.

The catheter worked well, with no reported issues with circuit pressures, bleeding, or displacement. The patient tolerated therapy, but, despite renal stabilization, care was ultimately redirected, and withdrawal of life-sustaining therapy occurred on DOL 4.

### Patient C

Patient C is a 35-week 6-day gestation male, birth weight of 3840 g, with suspected LUTO. APGARS were 5 and 8, but patient required resuscitation shortly and was placed on high-frequency oscillatory ventilation (HFOV), with required pressor support and anuria. He was transferred to COA on DOL 1. He had some urine upon placement of a Foley, however could not maintain appropriate fluid balance. Because he was unstable on maximal ventilatory support, the NICU NNP replaced his umbilical venous catheter (UVC) with the dELC and we initiated CVVH via Aquadex on DOL 4.

Blood flow was maintained at 40 mL/min with epoprostenol anticoagulation initiated at 4 ng/kg/min, then increased to 8 ng/kg/min after thirty min. CVVH dose of 24 mL/kg/h (207 mL/h/1.73 m^2^) was prescribed. The patient remained on therapy for 11 days. During that time, four Aquadex circuits were changed routinely at 72 h, as therapy will shut off after that time has elapsed. Two were changed early due to a patient procedure. Catheter function remained intact, with no mechanical or infectious complications.

After eleven days, the patient was taken to the OR for placement of a tunneled catheter for permanent CKRT access per institution practice. He remained on CKRT until he was able to have a PD catheter placed at age two months. He was transitioned to PD at three months. He was discharged home on PD at five months with a baseline oxygen requirement of 1 L/min nasal cannula.

### Patient D

Patient D is a 38-week 6-day gestation female, 2320 g at birth, born with APGARs of 6 and 8. She was diagnosed with bilateral multicystic dysplastic kidneys and no identifiable urinary bladder. At birth, she required tactile stimulation, continuous positive airway pressure (CPAP)/positive pressure ventilation (PPV), and then was ultimately intubated for respiratory failure. The baby remained anuric, so on DOL 1 she was transferred to COA for management of her congenital kidney failure.

A dELC was placed in the umbilical vein, swapping out the CVC that was placed at birth at the outside hospital, by the NNP. CKRT was initiated with CVVH via Aquadex on DOL 2 at a weight of 2.4 kg, with fluid overload of 3.4%. Blood flow was set at 40 mL/min (17.2 mL/kg/min) with epoprostenol anticoagulation, ramped to 8 ng/kg/min at 30 min per guidelines, and a prescribed CVVH dose of 24 mL/kg/h (171 mL/h/1.73 m^2^). The first circuit went down at 19.8 h due to increased withdrawal line pressures and clots found in the withdrawal lumen and was restarted 2 h later. Alteplase was injected into the catheter between circuit 1 and 2 and the next 2 circuits met goal, with routine changes around 72 h. Tunneled line placement was scheduled for the following day, DOL 10. The patient had an acute decompensation on DOL 10, requiring pressor support and a septic workup, requiring postponement of line placement. Also, the 4th circuit clotted on DOL 10 after 14 h and was restarted without issue. Catheter placement was associated with intermittent flow issues, thought to be related to anatomical positioning based on the patient’s intravascular volume and thrombi in the circuit rather than device failure.

The patient tolerated tunneled catheter placement on DOL 11 after septic workup remained negative. Gastric tube and PD catheter placement occurred at one month of age and PD was started three weeks after. The patient was discharged from the NICU at three months on PD and 0.5 L/min NC baseline oxygen requirement.

## Results

Four neonates with congenital or acquired kidney dysfunction were supported with CKRT within the first week of life. Gestational age ranged from 32.2 wga to 38.6 wga, with birth weights between 1.9 and 3.8 kg (Table 1). All patients required extracorporeal support for fluid overload or progressive kidney dysfunction, with three managed on the Aquadex and one on the PrisMax device. CVVH was the primary modality, with one patient managed on continuous CVVHDF. Anticoagulation strategies included epoprostenol (*n* = 3) and systemic heparin (*n* = 1).

**Table 1:** Clinical characteristics of patients including demographic and CKRT data

	Patient A (SFCH)	Patient B (COA 1)	Patient C (COA 2)	Patient D (COA 3)
Sex assigned at birth	Female	Male	Male	Female
Gestational age	32w4d	32w2d	35w6d	38w6d
Birth weight (kg)	1.94	2.00	3.84	2.32
Respiratory support	Conventional	Conventional	HFOV	CPAP
Etiology of kidney dysfunction	Severe LV dysfunction, acute tubular necrosis	Posterior urethral valves	Posterior urethral valves	Bilateral multicystic dysplastic kidneys
Age at KRT initiation (days)	7	1	4	2
Weight at KRT initiation (kg)	3.62	2.23	4.10	2.40
Fluid overload (%)	87.0	16.0	5.0	3.4
Device utilized	PrisMax	Aquadex	Aquadex	Aquadex
KRT modality	CVVHDF	CVVH	CVVH	CVVH
Blood flow (mL/min)	20	40	40	40
Blood flow (mL/kg/min)	10.0	20.0	10.4	17.2
Anticoagulation	Epoprostenol	Heparin	Epoprostenol	Epoprostenol
Effluent dose (mL/kg/h)	40	24	24	24
Effluent dose (mL/h/1.73 m^2^)	700	172	207	171
Days of catheter use	4	3	11	9
Total treatment time (hours)	81	63	259	217
Number of circuits	4	1	4	6
Catheter complications	Noted cracks in the hubs due to use of ClearGuard caps resulting in catheter replacement twice	None	None	Some issues with flow but likely due to placement itself, not catheter
CKRT outcome	Transitioned to non-tunneled catheter	Transitioned to comfort care	Transitioned to tunneled catheter in OR	Transitioned to tunneled catheter in OR
Patient outcome	Withdrawal of care	Withdrawal of care	Discharged on PD at 5 months	Discharged on PD at 3 months

*SFCH* Stead Family Children’s Hospital, *COA* Children’s of Alabama, *KRT* kidney replacement therapy, *CVVHDF* continuous veno-venous hemodiafiltration, *CVVH* continuous veno-venous hemofiltration, *CKRT* continuous kidney replacement therapy, *PD* peritoneal dialysis

Circuit performance varied across cases. Mean circuit life ranged from five to 71.7 h, median 54.9 h, with the longest circuit survival observed in patient C. Circuit complications were limited, though one patient (A) required catheter replacement due to cracks in hubs associated with ClearGuard caps. Catheter-related flow issues were noted in one term infant (D), though these were attributed to positioning rather than device malfunction. Two patients (A and B) ultimately transitioned to withdrawal of care, while two graduated from the NICU on PD.

## Discussion

This case series suggests that the use of the 6 Fr Nuwellis® dELC catheter placed in the umbilical vein is a feasible and effective option for establishing vascular access to initiate CKRT in neonates within the first week of life. Neonates requiring CKRT remain one of the most technically challenging patient populations due to limited vascular access options, small circulating blood volume, and hemodynamic fragility. In the USA, the lack of FDA-approved small dual lumen dialysis catheters has contributed to reliance on adapted adult or pediatric devices, which may compromise flow dynamics and increase risk of catheter dysfunction [4–6]. Our series adds to the small but growing body of evidence supporting innovative access strategies to overcome these limitations, particularly during the earliest neonatal period when alternative central access sites may not be available or safe.

Across the four neonates from two pediatric hospitals in this series, umbilical venous placement of the dELC provided sufficient blood flow to maintain continuous therapies using both Aquadex and PrisMax devices. Circuit survival times were clinically meaningful, with several circuits meeting or exceeding expected life goals per manufacturer guidelines. Notably, three of the four patients experienced no catheter-related mechanical complications, demonstrating that the coil-reinforced lumen design of the dELC catheter may reduce the risk of line collapse in low-pressure venous systems. The one case of catheter hub cracking was associated with post-placement handling rather than access failure, suggesting that workflow education may further reduce complications.

The umbilical vein offers distinct advantages for early neonatal access. It is readily available at birth, can be cannulated without sedation or fluoroscopy, and avoids the morbidity associated with internal jugular or femoral venous access in critically ill neonates. However, umbilical venous access has historically been avoided for prolonged extracorporeal therapy due to concerns for infection and catheter migration. In this series, no infectious complications attributable to the access site were identified despite therapy durations extending up to 11 days. Furthermore, the providers who placed the lines reported no issues with exchanging the CVC for the dELC and report no differences in placement compared to common CVCs used. While our findings suggest that umbilical placement may be more durable than previously assumed, careful insertion technique, secure line management, and early planning for transition to tunneled access remain essential. Safe and appropriate length of use of the access still remains unknown.

The outcomes in this series also underscore the importance of individualized clinical goals. Two patients ultimately transitioned to palliative care due to underlying disease severity rather than access or therapy failure. The remaining two progressed to PD and ongoing chronic kidney support, illustrating that early CKRT via umbilical access can serve as a bridge to longer-term renal replacement strategies. This aligns with emerging neonatal CKRT program models emphasizing staged therapy planning, multidisciplinary coordination, and proactive transition pathways.

This study has limitations. As a retrospective case series, the findings are descriptive and limited by small sample size. Additionally, catheter placement technique varied slightly between centers, with SFCH using interventional radiology to place the umbilical catheter and COA placing those catheters at the bedside, and circuit management protocols were not standardized. Future multicenter studies should assess standardized workflows and examine infection risk, flow performance, and circuit longevity in a larger population. Prospective data collection will also be important for regulatory advocacy to expand the availability of neonatal-specific extracorporeal devices in the USA.

In conclusion, this case series supports the use of the 6 Fr Nuwellis® dELC catheter placed via the umbilical vein as a viable temporary vascular access option for neonates requiring CKRT. This approach may expand access to lifesaving kidney support in the critical early neonatal period, particularly when traditional central venous access is not feasible. Continued innovation, shared clinical experience, and device-specific research are needed to advance safe and effective kidney support therapy for the most vulnerable patients.

## Supplementary Information

Below is the link to the electronic supplementary material.

ESM1 (PPTX 80.9 KB)

## Data Availability

The datasets generated during and/or analyzed during the current study are available from the corresponding author on reasonable request.
